# Supplementary material for: Quantifying the impact of physical distance measures on the transmission of COVID-19 in the UK
Source: BMC Med. 2020 May 7;18:124. doi: 10.1186/s12916-020-01597-8 (PMC7202922; doi:10.1186/s12916-020-01597-8)
Supplement: Supplementary file 1 — Additional file 1. CoMix study questions. [file 12916_2020_1597_MOESM1_ESM.pdf]

Covid 19 behaviour and contact survey

MAIN INTRO SCREEN

Thank you for agreeing to take part in this important research about the new coronavirus (COVID-19) pandemic. Please take the time to carefully read the information below and the detailed survey participation notice.

The survey is being run by Ipsos on behalf of a group of experts in mathematical and statistical modelling of infectious diseases and public health – London School of Hygiene and Tropical Medicine (LSHTM; United Kingdom), University of Hasselt (Belgium), University of Antwerp (Belgium), National Institute for Public Health and Environment agency (RIVM; the Netherlands), University of Bern (Switzerland), and the ISI Foundation Turin (Italy).

The research is part of a larger programme of work funded by the European Commission which aims to provide urgently needed answers about the epidemiological characteristics of COVID-19, the social dynamics of the outbreak, and the related public health preparedness and response to the ongoing pandemic, as well as to assess its economic impact. It will be used directly on active communication and interaction with policy makers/authorities, other scientific groups and the general public, to help minimise the COVID-19's public health, economic and social impact.

Taking part in this survey is completely voluntary, and you may refuse to do so. However, we would really value your support as the findings will provide invaluable information for decision making and strategies developed in the near future.

Ethics form for participants

I confirm that I am 18 years or older.

I confirm that I have read and understood the information for this study. I have had the opportunity to consider the information before taking part in this study.

I understand that taking part is voluntary, and that I am free to withdraw at any time without giving any reason and without any of my rights being affected.

I understand that relevant sections of anonymized data collected during the study may be looked at by authorised individuals from the University of Antwerp, London School of Hygiene & Tropical Medicine, National Institute for Public Health and Environment in the Netherlands, University of Bern, and the ISI Foundation Turin.

I understand that ANONYMIZED data about me may be shared via a public data repository and that I and any of my household members or contacts will not be identifiable from this information.

I understand that this is a longitudinal study, and that I will be invited to complete multiple questionnaires over the course of the ongoing epidemic.

1. Agree to take part
2. Do not agree to take part

Demographics

Q1 What is your age in years?
YEAR/MONTH

. Which of the following describes how you think of yourself?
1. Male
2. Female
3. In another way
4. Prefer not to answer

Participants in the UK ONLY

Q3. What is your current employment status?
1. 1 Employed full-time (34 hours or more)
2. 2 Employed part-time (less than 34 hours)
3. 3 Self employed
4. 4 Unemployed but looking for a job
5. 5 Unemployed and not looking for a job
6. 6 Full-time parent, homemaker
7. 7 Retired
8. 8 Student/Pupil
9. 9 Long-term sick or disabled

Are you the one in your household who has the highest income? [person with the largest income from employment, pensions, state benefits, investments or other sources]

1. 1 Yes
2. 2 Yes, together with another household member
3. 3 No

What is your occupation ? If retired or unemployed, please indicate the category closest to your previous occupation.

What is the occupation of the person with the highest income? If retired or unemployed, please indicate the category closest to his/her previous occupation.

What is your highest level of education attained?

1. 1 Without a diploma or primary education
2. 2 General lower secondary education (first 3 years completed)
3. 3 Technical, artistic or professional lower secondary education (first 3 years completed)
4. 4 General upper secondary education (6 years completed)
5. 5 Technical or artistic upper secondary education (6 years)
6. 6 Professional upper secondary (6 years)
7. 7 Higher education: graduat, candidature, bachelor
8. 8 University education: bachelor's degree, post-graduate, master's degree
9. 9 Complementary master
10. 10 Doctorate

What is the highest education level of the main earner?

1. 1 Without a diploma or primary education
2. 2 General lower secondary education (first 3 years completed)
3. 3 Technical, artistic or professional lower secondary education (first 3 years completed)
4. 4 General upper secondary education (6 years completed)
5. 5 Technical or artistic upper secondary education (6 years)
6. 6 Professional upper secondary (6 years)
7. 7 Higher education: graduat, candidature, bachelor
8. 8 University education: bachelor's degree, post-graduate, master's degree
9. 9 Complementary master
10. 10 Doctorate

What is the occupation of the main earner?

Participants in Belgium ONLY

What is the profession that the main earner last exercised?

Participants in the Netherlands ONLY

What is your occupation?

What is the occupation of the person with the highest income?

Q15. Are you currently pregnant?

1. 1 Yes
2. 2 No
3. 3 Prefer not to answer

What is the COMBINED TOTAL ANNUAL INCOME (pre-tax, pensions, or national insurance deductions) earned by all members of your family? Here, family is defined as everyone related by marriage, civil partnership, birth, or adoption living at the same address. Please include all your income sources: salaries, scholarships, pension and Social Security benefits, dividends from shares, income from rental properties, child support and alimony etc.

1. 1 Under £5,000
2. 2 £5,000 - £9,999
3. 3 £10,000 - £14,999
4. 4 £15,000 - £19,999
5. 5 £20,000 - £24,999
6. 6 £25,000 - £34,999
7. 7 £35,000 - £44,999
8. 8 £45,000 - £54,999
9. 9 £55,000 - £64,999
10. 9 £65,000 - £99,999
11. 10 £100,000 or more
12. 11 Prefer not to answer

Q20. Not including you, how many other people live in your household? By household, we mean anyone living at the same address as you, that you share a kitchen with.

None
1
2
3
4
5
6
7
8
9
10
11 or more

Q20a . Which of the following best describes your household?

2. Two or more non-family adults
3. Couple with dependent children
4. Couple with independent children only
5. Couple with no children
6. Lone parent with dependent children
7. Lone parent with independent children only

8. Households containing two or more families
9. Other

QNAME
Q21. Please write the nickname of each other person in your household.
Note that this nickname is only needed to make it easier for you to complete the survey, so please pick a nickname that will help you identify each household member later in the questionnaire. Nicknames are not visible to anyone outside of this survey.
1. NAME1
2. NAME2
3. NAME3
etc.

Q23. Which of the following age groups do they fit into?

1. Under 1
2. 1-4
3. 5-9
4. 10-14
5. 15-19
6. 20-24
7. 25-34
8. 35-44
9. 45-54
10. 55-64
11. 65-69
12. 70-74
13. 75-79
14. 80-84
15. 85+
16. Don’t know
17. Prefer not to answer

Q24. As far as you know, which of the following describes how [NAME] thinks of themselves?
1. 1 Male
2. 2 Female
3. 3 In another way
4. 4 Prefer not to answer
5. 5 Don’t know

Q25. What is [NAME]’s current employment status?
1. 1 Employed full-time (34 hours or more)
2. 2 Employed part-time (less than 34 hours)
3. 3 Self employed
4. 4 Unemployed but looking for a job
5. 5 Unemployed and not looking for a job

6. 6 Full-time parent, homemaker
7. 7 Retired
8. 8 Student/Pupil
9. 9 Long-term sick or disabled

Q26. Does [NAME] attend any of the following as a pupil or student?
1. Nursery or pre-school
2. School
3. Further education, e.g. college
4. Higher education, e.g. university
5. None of the above
6. Don't know
7. Prefer not to answer

Q27. Is [NAME] currently pregnant?
1. Yes
2. No
3. Don't know
4. Prefer not to answer

Q28. Are you or any other household member in a high-risk group under which the annual influenza vaccine would usually be offered by the NHS?
High risk groups include individuals with: chronic respiratory disease, chronic heart disease, chronic kidney disease, chronic liver disease, chronic neurological disease, diabetes (all types), immunosuppression (due to disease or treatment), asplenia or dysfunction of the spleen, class III obesity (BMI $\geq$ 40), and pregnant women.

0. Yourself
1. Name 1
2. Name 2
etc.

COLUMNS:
1. Yes
2. No
3. Don't know
4. Prefer not to answer

Symptoms
SYMPTOMS
Q29. Have you, or anyone else in your household, had any of the following symptoms in the last seven days?

ROWS:
0. Yourself
1. Name 1

2. Name 2
etc.

COLUMNS:
1. Fever or high temperature
2. A cough that has lasted for at least several hours
3. Shortness of breath
4. Aches and pains, e.g. in back, neck, shoulders or joints
5. Blocked nose
6. Sore throat
7. Feeling unusually tired
8. None of these
9 Don’t know
10. Prefer not to answer

Q30. Have you, or anyone else in your household, done any of the following for these symptoms?
ROWS:
0. Yourself
1. Name 1
2. Name 2
etc.

COLUMNS:
1. Phoned NHS 111 or used NHS 111 online service
2. Phoned a GP practice/GP out of hours service
3. Visited a GP practice/GP out of hours service
4. Visited a walk-in centre, urgent care centre, urgent treatment centre or minor injuries unit
5. Visited Accident & Emergency (A&E)
6. Visited a testing location somewhere different to these services
7. Been admitted to hospital
8. Don’t know
9. None of these
10. Prefer not to answer

Q31. You said that have/has [*INSERT SERVICE FROM Q30*]. When did [you/they] do that? If you don’t know exactly, please provide an approximate date.
Please use the format “DD/MM"
1. Don’t know
2. Prefer not to answer

Q32. Have you, or any other household member, ever been tested for Coronavirus (Covid-19)?

1. Yes
2. No

Q33. Who has been tested for Coronavirus (Covid-19)?

ROWS:
0. Yourself
1. Name 1
2. Name 2
etc.

COLUMNS:
1. Tested and the test showed I/they have Coronavirus
2. Tested, and the test showed I/they do not have Coronavirus
3. Yes, and I'm still waiting to hear the result
4. Not tested
5. Don't know
6. Prefer not to answer

Q34. To the best of your knowledge, do you think you or anyone else in your household have been in direct contact with someone who has Coronavirus (Covid-19) in the last seven days, or know someone close to them who has Coronavirus (Covid-19)?

ASK FOR SELF AND EACH PERSON NAMED AT Q21
ROWS:
0. Yourself
1. Name 1
2. Name 2
etc.

COLUMNS:
1. Yes, currently infected
2. Yes, passed away
3. Yes, recovered
4. No
5. Don't know
6. Prefer not to answer

Attitudes

Q35. To what extent do you agree or disagree with each of the following statements?

1. Strongly agree
2. Tend to agree
3. Neither agree nor disagree
4. Tend to disagree

5. Strongly disagree
6. Don't know

a) Coronavirus would be a serious illness for me

b) I am likely to catch coronavirus

c) If I don't follow the government's advice, I might spread coronavirus to someone who is vulnerable

Q36. How effective, if at all, do you think each of the following are at slowing the spread of coronavirus?

1. Very effective
2. Fairly effective
3. Not very effective
4. Not at all effective
5. Don't know

1. Reducing the number of people you meet
2. Staying at home for 7 days if you have a mild symptom such as a mild cough
3. Staying at home for 7 days if you have more severe symptoms such as a severe cough or a high temperature
4. Avoiding crowded places
5. Stay at home for 14 days if anyone other than yourself in your household has mild symptom such as a mild cough
6. Stay at home for 14 days if anyone other than yourself in your household has severe symptoms such as a cough or a high temperature
7. School closures
8. Closing bars, restaurants, cinemas etc.
9. Banning the use of public transport
10. Banning international travel into [the UK/Belgium/ the Netherlands]
11. Banning travel within [the UK/Belgium/ the Netherlands]

Q37. How confident are you, if at all, that if you wanted to you could…?

1. Very confident
2. Fairly confident
3. Not very confident
4. Not at all confident
5. Don't know

1. Reduce the number of people you meet
2. Stay at home for 7 days if you have a mild symptom such as a mild cough
3. Stay at home for 7 days if you have more severe symptoms such as a severe cough or a high temperature
4. Avoid crowded places
5. Stay at home for 14 days if anyone other than yourself in your household has mild symptom such as a mild cough

6. Stay at home for 14 days if anyone other than yourself in your household has severe symptoms such as cough or a high temperature
7. Not use public transport

Q38. To what extent do you agree or disagree with each of the following statements?

1. Strongly agree
2. Tend to agree
3. Neither agree nor disagree
4. Tend to disagree
5. Strongly disagree
6. Don't know

1. Other people I work with expect me to work, even when I am ill
2. If I could not work because of coronavirus, I would still get paid
3. If I had to isolate myself for 7 days because of coronavirus, someone else would be able to look after my children
4. If I had to isolate myself for 7 days, this would cause problems for other people who I don't know
5. I have enough food and supplies to last for 7 days, if I had to isolate myself

Behaviour

Q39. You may have been asked or decided to participate in different responses to coronavirus (covid-19).
Thinking about the last seven days, please select the appropriate response for each of the interventions listed below.

INTER In the last seven days, [*IF FOR SELF –* have you] [*IF FOR NAME 1/2 ETC AT Q21 –* has NAME] been asked to…

ROWS:
1. Quarantine [yourself/themselves]
Quarantine is the act of staying at home after a potential exposure to an infected case. If you are in quarantine, you can leave the house, but limit your movements.

2. Isolate [yourself/themselves]
Isolation is the act of separating yourself from people who are not infected, including any household members. You can be in isolation in your house or in a health facility.
3. Work from home due to coronavirus or limit your/their time at your/their workplace
4. Limit [your/their] time at the [university or college] OR [pre- school or nursery] OR [school] [due to coronavirus (covid-19)

COLUMNS:
1. Yes
2. No
3. Not applicable
4. Don't know
5. Prefer not to answer

Q40. In the last seven days, has…
ROWS:
1. [Your/ NAME's] workplace been closed due to coronavirus (covid-19) for at least one day 2. [Your/-NAME's] university or college] OR [pre-school or nursery] OR [school] been closed for at least one day

COLUMNS:
1. Yes
2. No
3. Not applicable
4. Don't know
5. Prefer not to answer

Q41. In the last seven days, [*IF FOR SELF –* have you] [*IF FOR NAME 1/2 ETC AT Q21 has NAME*]…
ROWS:
1. Been in quarantine for at least one day
Quarantine is the act of staying at home after a potential exposure to an infected case. If you are in quarantine, you can leave the house, but limit your movements.
2. Been in isolation for at least one day
Isolation is the act of separating yourself from people who are not infected, including any household members. You can be in isolation in your house or in a health facility.
3. Not been to your/their workplace for at least one day due to coronavirus (covid-19
4. Not attended [university or college] OR [pre-school or nursery] OR [school] due to coronavirus (covid-19) for at least one day

COLUMNS:
1. Yes
2. No
3. Not applicable
4. Don't know
5. Prefer not to answer

Q42. You said that [you have/NAME has] been in quarantine for at least one day. When did [you/they] start the quarantine?
1. INSERT DATE
2. Don't know
3. Prefer not to answer

Q43. And when did [you/NAME] finish the quarantine?
1. INSERT DATE
2. I am/they are still in quarantine
3. Don't know
4. Prefer not to answer

Q44. You said that [you have/NAME has] have been in isolation for at least one day. When did [you/they] start isolating?
1. INSERT DATE 2. Don't know
3. Prefer not to answer

Q45. And when did [you/NAME] finish isolating?
1. INSERT DATE
2. I am/they are still isolating
3. Don't know
4. Prefer not to answer

Q46. You said that [your/NAME's] [workplace] OR [university or college] OR [pre-school or nursery] OR [school] was closed due to coronavirus (covid-19) for at least one day in the last seven days. Please select the date when it was first closed.
1. INSERT DATE
2. Don't know
3. Prefer not to answer

Q47. And when did it open again?
1. INSERT DATE 2. It is still closed
3. Don't know
4. Prefer not to answer

Q48. You said that [you/NAME] did not attend [the workplace] OR [university or college] OR [pre-school or nursery] OR [school] due to coronavirus (covid-19), but it was not closed. For the last seven days, please select the days when [you/NAME] did not go to [the workplace] OR [university or college] OR [pre-school or nursery] OR [school] due to coronavirus (covid-19). Only indicate those days where [you/NAME] would normally have gone there.
1. SHOW DATES FOR LAST SEVEN DAYS
2. Don't know
3. Prefer not to answer

Q48A.
What was your main reason for not attending [the workplace] OR [university or college] OR [pre-school or nursery] OR [school] 7 day isolation due to you having symptoms that may be coronavirus

4. 14 day quarantine due to someone else in your household having symptoms that may be coronavirus, or due to contact with a known coronavirus case
5. Other illness (not coronavirus) within the household, (including yourself
6. Caring for someone outside of the household who has been confirmed to have coronavirus (COVID-19)
7. Caring for someone outside of the household who has not been confirmed to have coronavirus (COVID-19)
8. At least one child in my household is home due to school closure
9. Other

Q49. You said that [you/NAME] did not work/visit your workplace for at least one day due to coronavirus (covid-19) or that this was not applicable. Did this have a negative impact on your household income?
1. No, [I/NAME] was able to work from home
2. No, [I/NAME] was able to take carer leave
3. No, but [I/NAME] had to take annual leave
4. No, as [I/NAME] was fully compensated by [my/their] employer
5. No, as [I/NAME] was fully compensated by the government
6. Yes, but [I/NAME] received partial compensation by [my/their] employer
7. Yes, but [I/NAME] received partial compensation by the government
8. Yes, and [I/NAME] received no compensation for [my/their] lost income [SINGLE CODE ONLY]
9. Other (please specify)
10. Don't know
11. Prefer not to answer

Q50. You said that [NAME's] [pre-school or nursery] OR [school] was closed for at least one day due to coronavirus (covid-19). When this happened, who looked after the child/children?
CHILDCARE2
1. A parent, who is unemployed
2. A parent, who was working from home
3. A parent, who works part-time
4. A parent, who took annual leave
5. A parent, who took carer leave
6. A parent, who took unpaid leave
7. A sibling
8. Grandparent(s)
9. A baby sitter, childminder, au pair or nanny (paid)
10. A baby sitter, childminder, au pair or nanny (unpaid)
11. A neighbour, friend, uncle, or aunt
12. People at the school, as my child was eligible for childcare at the school
13. Not required
14. Other (please specify)

Q51. You said that [NAME] did not attend [pre-school or nursery] OR [school] for at least one day due to coronavirus (covid-19). When this happened, who looked after the child/children?
1. A parent, who is unemployed
2. A parent, who was working from home
3. A parent, who works part-time
4. A parent, who took annual leave
5. A parent, who took carer leave
6. A parent, who took unpaid leave
7. A sibling
8. Grandparent(s)
9. A baby sitter, childminder, au pair or nanny (paid)
10. A baby sitter, childminder, au pair or nanny (unpaid)
11. A neighbour, friend, uncle, or aunt
12. Not required
13. Other (please specify)

Q52. Did you visit, or intend to visit, any of the following events or locations in the last seven days?

ROWS:
a) Pub, bar or café
b) Restaurant
c) Cinema
d) Supermarket or other shop for food or groceries
e) Religious gathering
f) Sporting event (as participant), e.g. weekly tennis practice
g) Sporting event (as attendee), e.g. a football match
h) Indoor location, where over 100 people were present,
I) Outdoor location, where over 100 people were present

COLUMNS:
1. Yes, I visited this event or location
2. I intended to visit but it was cancelled because of the coronavirus (covid-19) epidemic
3. I intended to visit but chose not to go because of the coronavirus (covid-19) epidemic
4. I intended to visit but I had to cancel/it was cancelled for reasons unrelated to the coronavirus (covid-19) epidemic
5. No, I did not visit or intend to visit this event or location

Q53. You said that you intended to visit [a INSERT EVENT OR LOCATION] but it was cancelled because of coronavirus / but chose not to go because of coronavirus / but you had to cancel or it was cancelled for reasons unrelated to coronavirus]. How many times did that happen in the last seven days?
1. INSERT NUMBER OF TIMES
2. Don't know

Individual preventive measures

Q54. Did you use a face mask yesterday?
1. Yes

2. No

Q55. For how long did you wear a face mask in total?
Provide an approximation of the total duration.
INSERT NUMBER OF HOURS
INSERT NUMBER OF MINUTES

Q56. Where did you use your face mask?

1. Everywhere outside my house
2. When walking on the street
3. When cycling
4. On public transport
5. In supermarkets/shops
6. In cinema/bar/restaurant
7. At home
8. At work/school/college/university
9. Other (please specify)

Q57. How many times did you wash your hands with soap in the last three hours?
INSERT NUMBER OF TIMES (RANGE FROM 0-25)

Q58. How many times did you use hand sanitizer in the last three hours?
INSERT NUMBER OF TIMES (RANGE FROM 0-25)

Q59. Did you travel on any public transport yesterday?

1. No*]*
2. Train/tube
3. Bus/tram
4. Taxi, Uber, or similar ride-hailing app
5. Aeroplane

Q60. And approximately how long did you spend on the in total?
INSERT NUMBER OF HOURS
INSERT NUMBER OF MINUTES

Contact survey

We will now ask you to remember who you have been in contact with yesterday, between 5am yesterday and 5am today. We are only interested in direct contacts, which are **people who you met in person** and with whom you exchanged at least a few words, or with whom you had physical contact (e.g. a handshake, embracing, kissing, contact sports).
Note that if you only spoke to someone over the phone or internet, they should not be included in this section.

CONTACT1
Q62. Which of the following people did you have direct contact with in person, between 5am yesterday and 5am today, in person?

ROWS:
INSERT ALL NAMES FROM Q21

COLUMNS:
1. Yes
2. No

Q63. And what other people did you have direct contact with in person, between 5am yesterday and 5am today? Please think about anyone else you had direct contact with. This could include friends, family, work colleagues, or people you spoke to in shops and so on.

The order in which you give these names does not matter. However, it is easiest to give them in chronological order, e.g. when I woke up, I saw Peter and Naomi at breakfast. I then drove to my work, where I met with Jack, Deborah, and two clients. On my way back home, I stopped at a petrol station, where I had a brief chat with the shop assistant. Etc.

Please write the nickname of each person you had direct contact with. Note that this nickname is only needed to make it easier for you to complete the survey, so please pick a nickname that will help you identify each contact later in the questionnaire. Nicknames are not visible to anyone outside of this survey.

Q66. Which of the following age groups does NAME fit into? Please give an estimate if you are not sure

1. Under 1
2. 1-4
3. 5-9
4. 10-14
5. 15-19
6. 20-24
7. 25-34
8. 35-44
9. 45-54
10. 55-64
11. 65-69
12. 70-74
13. 75-79

14. 80-84
15. 85+
16. Don’t know
17. Prefer not to answer

Q67. As far as you know, which of the following describes how [NAME] thinks of themselves?

1. 1 Male
2. 2 Female
3. 3 In another way
4. 4 Prefer not to answer
5. 5 Don’t know

Q68. What is [NAME]’s relationship to you?
1. They are a family member who is not in my household
2. They are someone I work with
3. They are someone I go to school, college or university with
4. They are a friend
5. Other
6. Prefer not to answer

Q69. Before the coronavirus epidemic started, how often did you usually have direct contact with [*NAME*]?
A direct contact is when you **meet with this person in person** and when you exchange at least a few words, or when you have physical contact (e.g. handshake, embracing, kissing, contact sports).
Please do not include times that you speak to them over the phone or internet.

1. Every day or almost every day
2. About once or twice a week
3. Every 2-3 weeks
4. About once per month
5. Less often than once per month
6. Never met them before
7. Prefer not to answer

Q70. When you had direct contact with [NAME] yesterday, did you have…?
1. Physical contact (any sort of skin-to-skin contact such as e.g. hand shaking, embracing or kissing)
2. Non-physical contact (you did not touch the person)
3. Prefer not to answer

Q71. And where did you have direct contact with [NAME]?

1. At home
2. At someone else's house
3. At work
4. At a place of worship
5. On public transport
6. At school, pre-school, or nursery
7. At a supermarket, grocery store, or market
8. At a shop
9. At a place of entertainment such as a restaurant, bar, cinema
10. At a place for sports such as a gym or sports club/match
11. Outside, for example in a park, on the street or in the countryside
12. Somewhere else (please specify)

Q72. Please estimate the total amount of time you spent with [NAME] in person yesterday.

INSERT HOURS
INSERT MINUTES

Q73. Was the time you spent with [NAME] yesterday inside or outside?
Inside
Outside
