# Supplementary material for: Quantifying the impact of physical distance measures on the transmission of COVID-19 in the UK
Source: BMC Med. 2020 May 7;18:124. doi: 10.1186/s12916-020-01597-8 (PMC7202922; doi:10.1186/s12916-020-01597-8)
Supplement: Supplementary file 3 — Additional file 3. CoMix additional analyses. [file 12916_2020_1597_MOESM3_ESM.pdf]

Supplemental Material

Survey Recruitment

Data was collected on behalf of our study team by Ipsos, an international market research company. Participants are recruited by Ipsos from a variety of sources to create panels that are representative of the population in which they are recruited. Ipsos primarily recruits through social networks, allowing them to target hard to recruit populations, and includes providing participant-relevant incentives for completing surveys. Other methods for recruitment include email lists, banners, website and text ads, co-registration, and search engine marketing. When necessary, they also partner with thoroughly vetted third party recruiters. Ipsos limits the amount of surveys each participant is able to complete in a given time period, and uses algorithms to detect fraud and remove users from the survey in real-time.

For this survey, Ipsos recruited adults (ages 18 years and older) in census representative age bands. We compare our participants to census figures by age, gender, and household size.

Reproduction Number Distribution

Search terms and Results

Pubmed was searched using the terms “(2019 nCoV OR COVID) AND (reproduction number OR reproductive number OR severity OR incubation OR serial OR fatality)”. MedRxiv was searched with the terms “COVID OR ncov OR cov OR coronavirus OR SARS-cov-2 OR Novel coronavirus” with the last search on 15 March 2020. Both search terms were broad to include a range of epidemiological characteristics and clinical indicators as part of a wider data extraction effort. In addition, references of relevant publications were scanned for additional sources, and data was retrieved from the Midas Network. The CMMID COVID-19 Student group participated in the search and data extraction.

The search resulted in 49 estimates of the reproduction number using case data from China, Italy, South Korea, Singapore, Iran, and global cases. The central estimate of the reproduction number ranged from 0.3 to 7.05. The uncertainty intervals ranged from 0.17 to 8.46.

Methods

The studies were ranked from zero to five by modelling experts for quality and type of data collection, method and application of method, and plausibility of the estimate. Only early outbreak data was included to remove estimates that were likely to have been affected by public health interventions or independent behavior changes. Only studies with a quality score above one were included.

To parameterize each of the included distributions, we used the Nelder-Mead optimization algorithm to identify the PERT distribution (a scaled beta distribution, characterised by a minimum value, a maximum value, and a modal value) that uniquely fit the central estimate and uncertainty interval reported by each study, using the mc2d[1] and nloptr[1] R packages. The PERT distribution was used because it is able to capture skewed bell-shaped distributions. As most studies reported the 95% confidence interval and some studies did not report the interval type, all uncertainty intervals were assumed to represent the 95% confidence interval. Each parameterized distribution was then sampled 10,000 times to produce the final consensus distribution. As all of the included studies had been assigned a score of two or three, weighting the estimates made no difference, so no weighting was applied to the final distribution.

Value of the distribution

The weibull, gamma, and normal distributions fit to the combined data. See Table 2 for the fitted parameters, and Figure S1 for the density plots. We used the normal distribution with a mean of 2.6 and a standard deviation of 0.54.

Included Studies

Table S1. Table of included studies

First Author [number]	Location	Central Estimate	Uncertainty Interval - Low	Uncertainty Interval - High	Central Estimate Type	Quality Score
Riou, J[2]	China	2.2	1.4	3.8	NA	3
Imai, N[3]	China	2.6	1.5	3.5	mean	2
Read, J[4]	NA	3.11	2.39	4.13	mean	2
Zhao, S[5]	China	2.24	1.96	2.55	mean	2
Liu, T[6]	NA	2.9	2.32	3.63	NA	2
Chinazzi, M[7]	NA	2.4	2.2	2.6	mean	2
Wu, T[8]	NA	2.68	2.47	2.86	NA	2
Jung, S[9]	China	2.1	2	2.2	NA	2
Jung, S [2]	China	3.2	2.7	3.7	NA	3

Zhuang, Z[10]	Italy	2.6	2.3	2.9	mean	3
Zhuang, Z [2][10]	Italy	3.3	3	3.6	mean	3
Zhuang, Z [3][10]	South Korea	2.6	2.3	2.9	mean	3
Zhuang, Z [4][10]	South Korea	3.2	2.9	3.5	mean	3
Chong, K[11]	Zhejiang, China	2.08	1.49	2.72	mean	2
Chong, K [2][11]	Zhejiang, China	1.88	1.38	2.41	mean	2
Li, Qun[12]	Wuhan, China	2.2	1.4	3.9	unspecified	2
Abbott, S[13]	*NA*	2.5	2	3	NA	2

Figure S1. Density plots for the combined reproduction number (R0) and fitted distributions

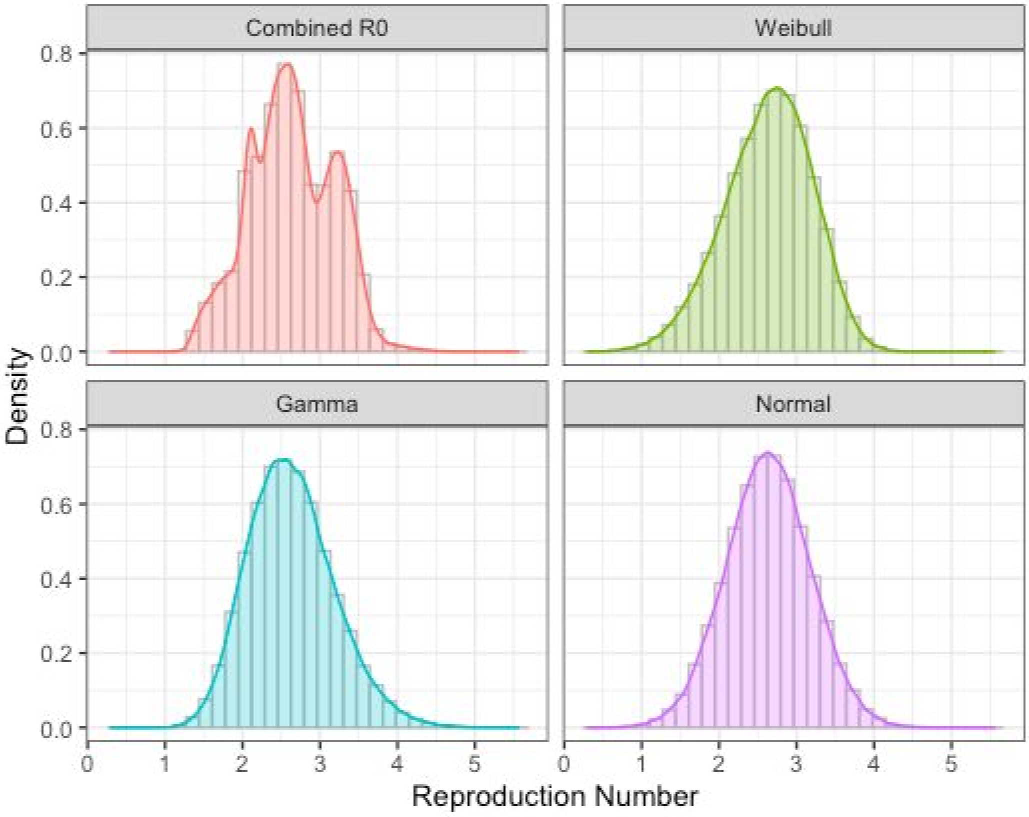

Table S2. Parameters of the fitted distributions

Distribution	Parameter 1 Type	Parameter 1	Parameter 2 Type	Parameter 2
Weibull	shape	5.39	scale	2.85
Gamma	shape	22.44	rate	8.52
Normal	mean	2.63	sd	0.54
